# FatPlants 2.0: an AI‐powered platform integrating plant lipid genes, pathways, and literature

**DOI:** 10.1111/tpj.71117

**Published:** 2026-09-15

**Authors:** Yongfang Qin, Congyu Guo, Minseong Kim, Yichuan Zhang, Muhammad Azam, Tingyuan Xiao, Chunhui Xu, Basil Shorrosh, Timothy P. Durrett, Doug K. Allen, Trupti Joshi, Edgar B. Cahoon, Jay J. Thelen, Dong Xu

**Affiliations:** ^1^ Department of Electrical Engineering and Computer Science University of Missouri Columbia Missouri 65201 USA; ^2^ Christopher S. Bond Life Sciences Center University of Missouri Columbia Missouri 65211 USA; ^3^ Department of Life Science University of Seoul Seoul 02504 Republic of Korea; ^4^ Health Informatics Institute University of South Florida Tampa Florida 33612 USA; ^5^ Department of Biochemistry and Molecular Biophysics Kansas State University Manhattan Kansas 66502 USA; ^6^ AI & Data Knowledge Engineering, Accenture Denver Colorado 80202 USA; ^7^ Donald Danforth Plant Science Center St. Louis Missouri 63132 USA; ^8^ Institute for Data Science and Informatics University of Missouri Columbia Missouri 65211 USA; ^9^ Department of Biomedical Informatics, Biostatistics and Medical Epidemiology University of Missouri Columbia Missouri USA; ^10^ Department of Biomedical Sciences Marshall University Huntington West Virginia 25755 USA; ^11^ Department of Biochemistry and Center for Plant Science Innovation University of Nebraska Lincoln Lincoln Nebraska 68588 USA; ^12^ Department of Biochemistry University of Missouri Columbia Missouri 65211 USA

**Keywords:** plant lipids, FatPlants, database, pathway analysis, AI chatbot, large language model, retrieval‐augmented generation (RAG)

## Abstract

Plant lipid research depends on accessible databases that connect genes, pathways, and prior literature. However, currently available resources are fragmented, species‐limited, and lack AI‐powered interfaces for integrated querying. FatPlants 2.0 addresses these issues by integrating ARALIP, PlantFADB, and new Cuphea/Pennycress experimental data into a unified platform with 14 000 genes/proteins, 110 pathways across 5 species, and 57 000+ curated publications. The platform features LipidBot, an AI agent enabling natural language queries via graph‐based pathway search and retrieval‐augmented generation‐powered literature retrieval. Users query complex relationships conversationally and receive answers with traceable citations. The graph database models 100+ biological pathways as queryable networks with large language model‐guided Cypher generation. By evaluating more than 1000 curated questions, LipidBot achieved 95% accuracy on pathway queries and 92% recall in literature retrieval using optimized embeddings. The tool demonstrated robust performance across factual, numerical, and multi‐hop queries on curated benchmarks. FatPlants 2.0 accelerates research by reducing the time spent on literature reviews. Database and AI agent freely available at https://fatplants.net with bulk downloads and quarterly updates.

## Significance Statement

Understanding plant lipid metabolism requires connecting genes, pathways, species, and experimental evidence that are currently scattered across multiple resources. FatPlants 2.0 unifies these resources in a single platform and introduces LipidBot, a retrieval‐augmented AI agent that combines knowledge graph querying with more than 57 000 plant lipid‐related publications to provide source‐traceable answers, support cross‐species comparisons, and accelerate biological interpretation and hypothesis generation.

## INTRODUCTION

Plant lipids are essential for membrane structure, energy storage, signaling, and stress response (Xu et al., 2023; Yang & Benning, 2018). Understanding lipid metabolism is crucial for crop improvement, biofuel production, and nutrition enhancement (Tirado et al., 2010). Recent advances in oilseed crops such as Cuphea for medium‐chain fatty acids (Graham, 1989) and pennycress, a winter cover crop with high oil content, highlight the need for comparative resources spanning diverse species (Ma et al., 2023), which are still largely unavailable.

Beyond limitations in data coverage, a fundamental challenge in plant lipid research lies in the fragmented and relational nature of biological knowledge (Xu et al., 2024). Lipid metabolic processes are inherently multi‐layered, involving coordinated interactions among genes, enzymes, metabolites, pathways, subcellular compartments, and environmental or developmental contexts (Xu et al., 2023). Evidence for these relationships is dispersed across independent studies and heterogeneous databases, impeding systematic knowledge integration and limiting our ability to answer cross‐pathway and cross‐species questions, such as how enzymes connect multiple lipid pathways or how findings from emerging oilseed crops relate to model systems.

Traditional database interfaces, which are primarily designed for keyword‐based searches or static pathway browsing, are poorly suited to address such relational and multi‐hop queries (Hogan et al., 2022). Users are often required to manually navigate between databases and publications, a process that is both time‐consuming and prone to omission, particularly when exploring complex cross‐pathway or cross‐species relationships (Gao, Xiong, et al., 2024). As a result, the rapidly expanding volume of lipid‐related data has not translated into proportional gains in biological insight (Marx, 2013).

Existing lipid community resources have significant gaps. ARALIP (Li‐Beisson et al., 2013) provides comprehensive, expertly curated lipid metabolic pathways for *Arabidopsis thaliana*; however, its single‐species focus limits comparative analyses across diverse plant taxa, and the resource is no longer actively maintained. PlantFADB (Ohlrogge et al., 2018) is designed for multi‐species plant fatty acids, but it lacks pathway integration. KEGG (Kanehisa et al., 2023) has broad coverage but is limited to primary literature and lacks a natural language (NL) query interface. LipidMaps (Conroy et al., 2024) is animal‐centric with minimal plant annotations. Consequently, lipid‐related knowledge remains dispersed, under‐integrated, and particularly sparse for emerging oilseed crops. SwissLipids (Aimo et al., 2015) advances reaction‐level precision by linking lipid structures to enzymatic reactions and UniProt protein identifiers, but its curation has focused predominantly on human, mouse, and yeast, leaving plant ceramides, glycolipids, and other plant‐specific lipid classes substantially underrepresented. MetaCyc (Caspi et al., 2020) provides experimentally supported reaction stoichiometry and pathway descriptions, but it is organized around reactions and metabolites rather than genes, making it poorly suited for gene‐centric queries. CLAIR (He et al., 2024) extends lipid gene coverage to nine major oilseed crops with RNA‐seq co‐expression data and improved gene set curation, but it does not include lipid structural data or reaction‐level biochemistry. AI‐assisted tools for efficient synthesis and exploration are largely absent.

Recent advances in large language models (LLMs) have demonstrated strong capabilities in NL understanding and question answering (Gao, Fang, et al., 2024; OpenAI et al., 2024); however, their application to biological knowledge exploration remains limited by hallucination, lack of traceability, and insufficient grounding in structured domain knowledge (Gao, Xiong, et al., 2024; Ji et al., 2023). Knowledge graph representations provide a complementary framework by explicitly modeling biological entities and their relationships, enabling transparent reasoning over complex networks and supporting complex, multi‐hop queries (Gao, Xiong, et al., 2024). Nevertheless, most existing plant lipid resources do not adopt graph‐based architectures or integrate LLMs in ways that preserve scientific rigor, interpretability, and citation provenance (Himmelstein et al., 2017).

To address these limitations, we developed FatPlants 2.0 as an integrated knowledge hub for plant lipid research. Building on FatPlants 1.0 (Xu et al., 2024), FatPlants 2.0 expands the platform's data content and analytical capabilities. It incorporates updated multi‐source plant lipid data, emerging oilseed datasets, a larger curated literature corpus, graph‐native pathway representation, and LipidBot, an AI‐assisted agent for source‐attributed natural language querying. An extended comparison with FatPlants 1.0 and related plant lipid resources is provided in Table S1. Together, these additions are intended to support comparative analysis, efficient knowledge exploration, and hypothesis generation in plant lipid biology.

## RESULTS

### Content

Data from six primary sources (Table 1) were integrated into FatPlants 2.0. From ARALIP (Li‐Beisson et al., 2013), we imported 23 Arabidopsis‐specific pathways comprising 811 genes, 378 enzymes, and 295 characterized mutants. PlantFADB (Ohlrogge et al., 2018) contributed 495 unique fatty acid structures spanning >9000 plant species across 231 families. Systematic searches of KEGG (Kanehisa, 2000), PMN (Hawkins et al., 2025), and MetaCyc (Caspi et al., 2020) databases using lipid‐related terms (“glycerolipid,” “phospholipid,” “fatty acid,” “sphingolipid,” “sterol”) yielded 100+ plant lipid metabolism pathways across four focal species: *A. thaliana*, *Aegilops tauschii*, *Camelina sativa*, and *Glycine max*. Each pathway includes complete reaction stoichiometry, EC classifications, and gene–enzyme associations. All pathway data were converted into a graph‐based representation where nodes represent biological entities and edges denote biochemical reactions, regulatory relationships, and genetic associations. The knowledge graph includes six primary node types: pathways, reactions, enzymes, orthologs, genes, and compounds. Edges represent biological relationships, including catalysis, regulation, substrate‐product transformations, and cross‐species orthologs. The database contains >4300 nodes and >10 700 relationships. Plant fatty acid literature was retrieved from PubMed and OpenAlex (Priem et al., 2022) using systematic keyword searches (Table S2). A two‐stage LLM‐based classification pipeline filtered for plant‐specific and lipid‐relevant studies, resulting in a curated corpus of over 57 000 publications for retrieval‐augmented generation.

**Table 1:** Database integration statistics

Database	Species	Pathways	Reactions	Genes	Enzymes	Ortholog	Compounds	Fatty acids	Publications
ARALIP	1	23	—	811	378	—	—	—	>500
PlantFADB	>9000	—	—	—	—	—	—	495	>3000
KEGG	4	76	484	1910	297	594	492	—	—
PMN	1	24	—	—	—	—	—	—	—
PubMed	—	—	—	—	—	—	—	—	12 364
OpenAlex	—	—	—	—	—	—	—	—	56 009
FatPlants 1.0	3	>40	—	—	>12 000	—	—	495	—
FatPlants 2.0	6^a^+ >9000^b^	>110	484	1910	>12 000	594	492	495	>57 000

^a^ Focal species for curated pathway data: *Arabidopsis thaliana*, *Aegilops tauschii*, *Camelina sativa*, and *Glycine max*. Cuphea and Pennycress represent emerging oilseed crops with experimental data.

^b^ PlantFADB fatty acid distribution data spans >9000 plant species across 250 families.

The platform integrates experimental data from emerging oilseed species, including Cuphea and pennycress, through curated community contributions. PacBio Iso‐Seq derived protein sequences are queried against the FatPlants lipid protein database using BLASTP (Altschul, 1997; Camacho et al., 2009), retaining matches with at least 70% sequence identity over a minimum alignment length of 100 amino acids.

### Platform features

**Figure 1:**
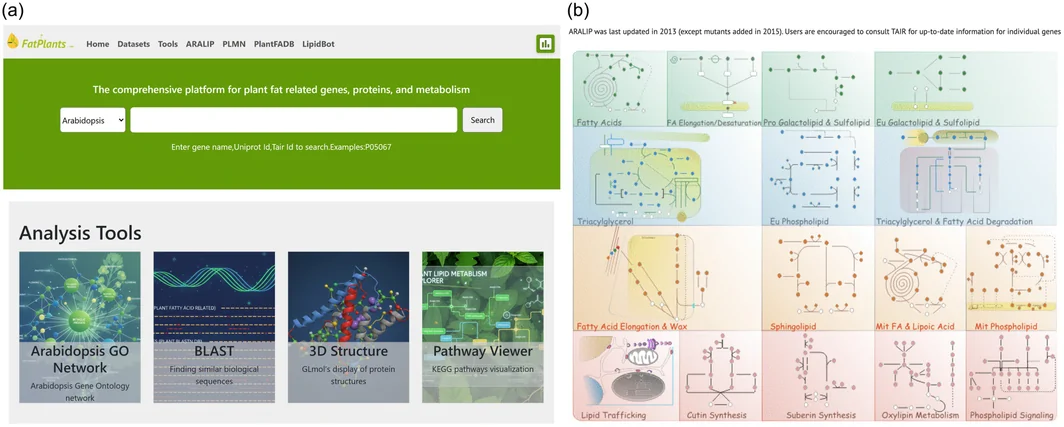
The overview of FatPlants. (a) Landing page of FatPlants, showing the primary search interface and the structured navigation menu. (b) ARALIP‐Curated Lipid Metabolic Pathways.

**Figure 2:**
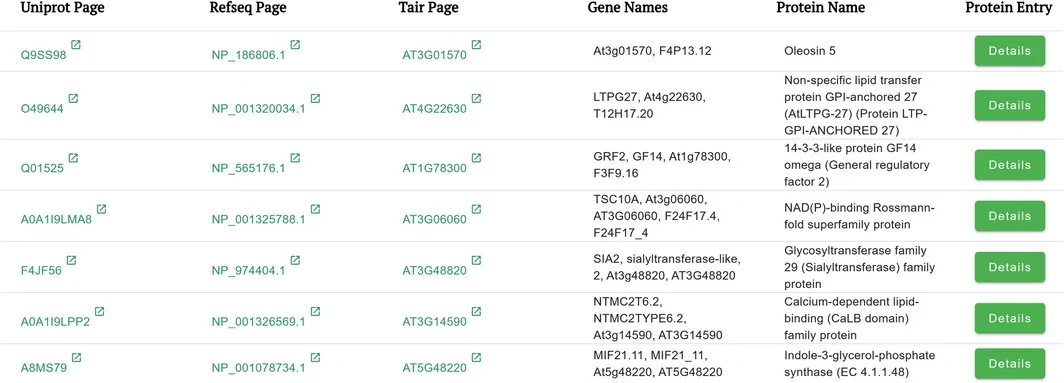
The information table displaying fatty acid‐related enzymes in *Arabidopsis thaliana*, with seven entries shown for illustration.

FatPlants 2.0 provides a user‐interactive web‐based interface optimized for the querying, browsing, and comparative analysis of plant lipid data. The platform is structured around discrete data modules, accessible via a primary search interface and a structured navigation menu (Figure 1a). To support high‐throughput analysis, we integrated four core computational tools, including a curated BLAST server containing >12 000 lipid‐related enzyme sequences. For each identified gene or enzyme, the platform renders a comprehensive data profile encompassing primary sequence data, cross‐species ortholog mappings, and multi‐source functional annotations (Figure 2).

We fully integrated the ARALIP database into the FatPlants framework, enabling seamless retrieval of curated lipid metabolism genes, enzymes, and pathways within a unified environment (Figure 1b). The platform currently hosts 24 high‐priority metabolic pathways curated from MetaCyc (Caspi et al., 2020). These pathways are rendered through an interactive graphical interface in which nodes and edges are hyperlinked to the original database entries, facilitating multi‐hop data exploration.

**Figure 3:**
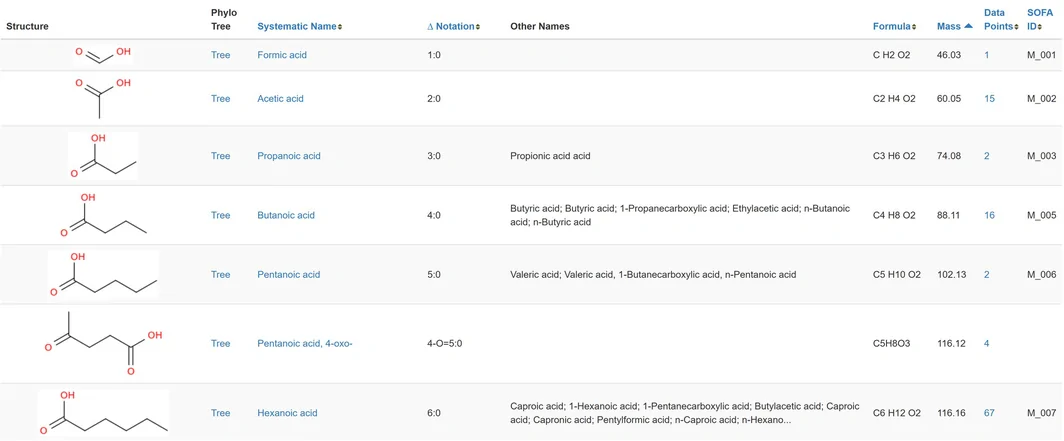
Overview of the display of fatty acid chemotaxonomic information in PlantFADB. All fatty acids are linked to supporting literature references, with seven entries shown for illustration.

The platform incorporates PlantFADB, a specialized resource dedicated to plant fatty acid diversity (Figure 3). PlantFADB allows researchers to investigate phylogenetic relationships across thousands of plant species and hundreds of distinct fatty acids. Feature sets include high‐resolution chemical structure visualization, quantitative fatty acid composition data, and total oil content metrics, all of which are cross‐referenced with primary literature.

**Figure 4:**
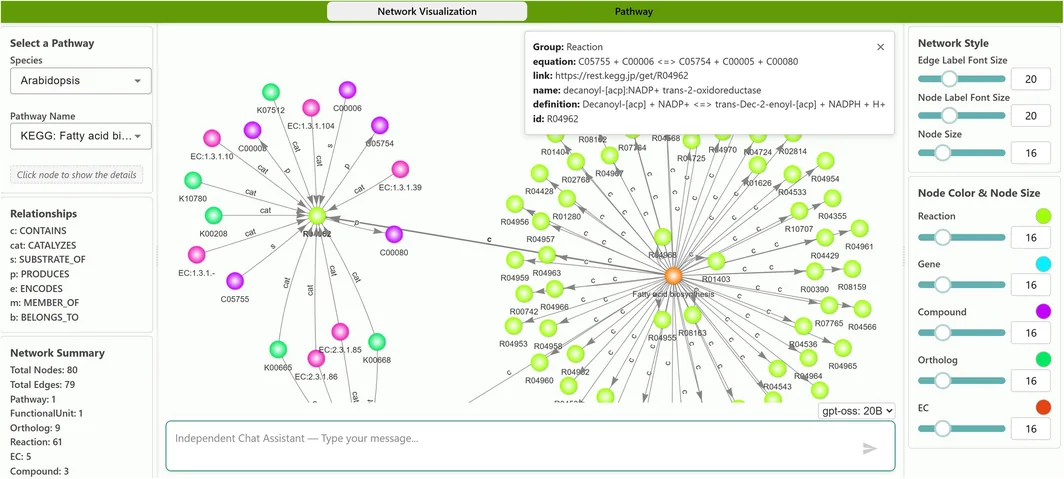
LipidBot interface enabling natural language query processing, with interactive visualization of pathway graph data.

To enhance data accessibility, we developed LipidBot (Figure 4), an AI‐driven agent that processes natural language queries. The system utilizes LLMs, including GPT‐based (OpenAI et al., 2025) and Llama (Touvron et al., 2023) architecture. LipidBot translates the user's NL into Neo4j Cypher queries to interrogate the underlying graph database, subsequently retrieving relevant biological data and associated citations. To ensure scientific rigor, all conversational outputs include traceable citations to original databases or peer‐reviewed publications.

All curated data are freely available through the web interface, including bulk downloads for enzyme, gene/mutation, and fatty acid distribution data, PNG/JPG exports of pathway diagrams, and the full graph database dump for local analysis. Downloads are accessible via individual data pages or a centralized Data Download page.

#### Visits statistics

FatPlants 2.0 recorded 2378 visits from 55 countries between July and December 2025, generating 10 856 total interactions (Table S3). The platform achieved a 32% return visitor rate, with returning users demonstrating substantially higher engagement (8.5 actions, 8.8 min per session) than first‐time visitors (2.7 actions, 1.6 min per session). The geographic distribution shows concentrated usage in China (30%) and the United States (30%), collectively accounting for 60% of traffic. Asian institutions represent the largest regional user base (48%), followed by North America (31%) and Europe (17%) (Figure S1).

### Case study using LipidBot

LipidBot accepts plain‐language questions about plant lipid metabolism and is designed to support biologically meaningful tasks in plant lipid research, including candidate gene prioritization, cross‐species reconstruction of biosynthetic pathways, and synthesis of regulatory evidence from dispersed literature. By linking structured FatPlants data with a curated plant lipid literature corpus, LipidBot connects genes, enzymes, pathways, species‐specific evidence, and published findings into interpretable evidence chains. The following use cases illustrate how this integrated framework supports the generation of biologically testable biological interpretations. In each case, the query was submitted exactly as written, and supporting citations were retrieved automatically from a curated corpus of over 57 000 plant lipid publications.

#### Use case 1: cross‐species gene targeting for high‐oleate soybean oil

To demonstrate LipidBot's ability to support targeted crop engineering, we designed a use case to identify soybean FAD2 paralogs relevant to oleic acid accumulation. A plant biochemist developing high‐oleate varieties submits the query: ‘Which soybean FAD2 paralogs are most strongly associated with oleic acid accumulation, and what Arabidopsis suppression studies support targeting their orthologs?’

LipidBot queries the pathway graph to retrieve FAD2 family members involved in fatty acid desaturation in soybean and then integrates the literature corpus to rank candidates by evidence strength. It identifies FAD2‐1a as the primary target: deletion of this gene in the X‐ray‐induced mutant M23 produces seed oil with approximately over 70% oleic acid, compared with roughly 20% in conventional lines, consistent with FAD2‐1a being the dominant enzyme converting oleic to linoleic acid in developing seeds (Pham et al., 2010; Sandhu et al., 2007). FAD2‐1b is flagged as the secondary target due to its predominant embryo expression during seed fill (Sandhu et al., 2007). For Arabidopsis validation, LipidBot retrieves functional evidence confirming that AtFAD2 manipulation directly governs the oleic/linoleic ratio and flags a co‐suppression risk under 35S‐driven overexpression, which was rescued only in the *rdr6* mutant background (Du et al., 2019; Li et al., 2025), recommending embryo‐specific promoters as a mitigation strategy.

This use case demonstrates LipidBot's capacity to combine pathway‐level gene retrieval with citation‐grounded literature synthesis into a unified, actionable response.

#### Use case 2: multi‐gene metabolic engineering for biofuel feedstock optimization

To demonstrate LipidBot's capacity for multi‐hop reasoning across species and pathway layers, we designed a use case centered on medium‐chain fatty acid (MCFA) engineering in Cuphea. A researcher investigating jet fuel feedstock production submits the query: ‘Which Cuphea genes involved in medium‐chain fatty acid accumulation have characterized orthologs in Arabidopsis, and what co‐expression or regulatory relationships connect them to triacylglycerol assembly pathways?’

LipidBot queries the pathway graph to retrieve Cuphea genes connected to MCFA biosynthesis and their mapped Arabidopsis orthologs, identifying CcFATB1, CcFATB2, KAS IV, and KAS A1 as linked to the FATB and KAS II families, respectively. LipidBot retrieves literature confirming the functional basis of these ortholog relationships: CcFATB1 and CcFATB2 have been shown to redirect fatty acid flux toward medium‐chain products when expressed in Arabidopsis seeds, with MCFA subsequently incorporated into TAG via endogenous Arabidopsis DGAT1 (Filichkin et al., 2006), and Cuphea ChKAS IV has been characterized as a medium‐chain‐specific condensing enzyme whose co‐expression with MCFA thioesterases enhances 8:0, 10:0, and 12:0 accumulation (Dehesh et al., 1998) in transgenic *Brassica* plants. LipidBot further retrieves evidence that co‐expression of a Cuphea MCFA thioesterase with AtWRI1 and AtDGAT1 substantially increased TAG accumulation in *Nicotiana benthamiana* leaves (Reynolds et al., 2015). The disruption of plastidial acyl‐ACP synthetases AAE15/16 further boosts MCFA accumulation in transgenic Arabidopsis seeds (Tjellström et al., 2013), flagging these as additional engineering targets.

This case illustrates LipidBot's ability to combine pathway‐level ortholog mapping with citation‐grounded mechanistic evidence to reconstruct the biosynthetic and regulatory architecture surrounding a multi‐gene engineering goal.

#### Use case 3: literature‐grounded regulatory synthesis across oilseed species

To demonstrate LipidBot's capacity for citation‐grounded literature synthesis, distinct from its pathway graph capabilities, we designed a use case focused on the transcriptional regulation of fatty acid biosynthesis. A researcher studying the conservation of seed oil regulatory networks submits the query: ‘What experimental evidence links WRI1 to fatty acid biosynthesis regulation across different oilseed species, and are there documented differences in its downstream targets between species?’

Unlike the preceding use cases, this query has no single pathway answer. It requires synthesis across species, experimental approaches, and decades of primary literature. LipidBot queries its curated corpus and returns a species‐stratified response with traceable citations. For Arabidopsis, LipidBot retrieves literature reporting that WRI1 directly activates fatty acid biosynthetic genes and upstream glycolytic genes, including FRK3 and PGI1, with AW box binding affinity confirmed across 11 Brassicaceae species (Kuczynski et al., 2022), and that WRI1 functions downstream of the master regulator LEC2 within a defined seed maturation regulatory hierarchy (Baud et al., 2007). For sunflower, LipidBot retrieves literature identifying a documented divergence: sunflower WRI1 targets a broad set of plastidial genes, including the pyruvate dehydrogenase complex, α‐CT, BCCP, ACPs, and FATA1, and binds a non‐canonical AW box motif with a distinct base bias not observed in Arabidopsis (Sánchez et al., 2022). Across soybean, cotton, rapeseed, and sesame, LipidBot retrieves comparative transcriptomic evidence consistent with a shared WRI1‐type regulatory module underlying oil accumulation in diverse crops (Chen et al., 2024).

This use case demonstrates that LipidBot's RAG‐powered literature retrieval complements its graph‐based pathway reasoning, enabling researchers to move fluidly from structured gene–enzyme–pathway queries to nuanced, citation‐supported synthesis of regulatory mechanisms across the oilseed literature.

### Evaluation framework and datasets

We evaluated FatPlants 2.0 LipidBot using a two‐part benchmark designed to assess targeted literature‐based question answering and structured pathway reasoning within the curated FatPlants knowledge framework. The purpose of this evaluation was not to position LipidBot as a replacement for broad literature search engines, but to assess whether it could reliably integrate plant lipid–specific evidence retrieval with graph‐based knowledge access, enabling users to move beyond simple database record retrieval toward biologically meaningful connections among genes, pathways, species, and supporting literature. The Question‐to‐Citation benchmark evaluates whether LipidBot can retrieve experimental and regulatory evidence from the plant lipid literature, supporting tasks such as gene‐function interpretation, candidate target validation, and evidence synthesis across studies. The NL‐to‐Cypher benchmark evaluates whether natural language questions can be translated into executable graph queries that retrieve genes, enzymes, compounds, orthologs, and pathway relationships. Together, these benchmarks connect technical performance metrics to biological use cases, including pathway reconstruction, cross‐species comparison, candidate gene prioritization, and evidence‐based hypothesis generation.

To ensure statistically robust performance estimates, the sample size was determined using the binomial proportion formula,

$$n=\frac{Z_{\alpha /2}^{2}\times p\times \left(1-p\right)}{E^{2}}$$

with $Z_{\alpha /2}=1.96\;\left(95\%\text{confidence level}\right)$, *p* = 0.5 (maximum variance assumption), and $E=0.05\;\left(\text{margin of error}\right)$, yielding a minimum required sample size of n≥384. Two datasets were constructed: an 800‐question Question‐to‐Citation benchmark and a 393‐question pathway NL‐to‐Cypher generation benchmark (Table S12).

#### Construction of a question‐to‐citation benchmark

The literature‐based Question‐to‐Citation benchmark comprises 800 questions stratified across eight predefined question categories (100 questions per category; Table S4), organized using Topic and Archetype Scaffolding to ensure balanced coverage.

Questions and corresponding expert‐curated reference answers were generated through a citation‐by‐citation workflow. For each source citation, an LLM was prompted to produce candidate questions via prompt‐guided, citation‐conditioned generation. To promote semantic reasoning rather than keyword matching, questions were intentionally designed to minimize lexical overlap with the source citation while retaining essential biological terms and entity names for precise interpretation. Questions were formulated by paraphrasing and substituting semantically equivalent expressions to preserve the original biological meaning and factual accuracy. The reference answer for each question is the unique identifier (ID) of the source citation from which the question was derived. Domain experts subsequently reviewed and validated all questions against the original literature, verifying that each question is unambiguously and exclusively answerable by its paired source citation before final inclusion in the benchmark.

#### Evaluation of structured pathway reasoning via a NL‐to‐Cypher benchmark

Structured pathway reasoning was evaluated using a 393‐question NL‐to‐Cypher benchmark. Queries were systematically stratified by reasoning complexity into three tiers: (1) Single‐Hop Factual Retrieval: Direct entity lookups or single‐step graph traversals. (2) Relational and Multi‐Hop Reasoning: Queries requiring two to four traversal steps, including cross‐pathway and cross‐species inference. (3) Robustness and Error Handling: Ambiguous or incomplete queries designed to assess error detection and recovery mechanisms.

#### Baseline comparisons and evaluation metrics

We compared LipidBot against two established baseline approaches representing complementary paradigms: (1) BM25 (Robertson & Zaragoza, 2009) and PubMedBERT‐Base (Gu et al., 2022), as retrieval‐focused baselines for unstructured literature question answering. (2) LangChain CypherQAChain (LangChain, 2025), as a representative NL‐to‐Cypher generation system for structured pathway querying.

#### Literature retrieval evaluation

For unstructured literature retrieval, we adapted the BioASQ (Krithara et al., 2023) evaluation framework. The system was evaluated on Factoid and Numerical questions using standard information retrieval metrics, including Precision@10, Recall@10, Hit Rate@10, Mean Reciprocal Rank (MRR@10), and normalized Discounted Cumulative Gain (nDCG@10). All systems were evaluated against the same document corpus, ensuring a fair and controlled comparison.

Within the curated Question‐to‐Citation benchmark, quantitative evaluation (Table 2) shows that LipidBot substantially improves retrieval coverage relative to lexical and domain‐specific transformer baselines. LipidBot achieved a Hit Rate@10 of 0.919, outperforming BM25 (0.884) and PubMedBERT‐Base (0.678), indicating more effective semantic retrieval for complex metabolic queries.

**Table 2:** Literature retrieval performance

Model	MRR@10	Hit Rate@10	Recall@10	Precision@10	nDCG@10
BM25	**0.809**	0.884	0.884	0.088	**0.827**
PubMedBERT (base)	0.511	0.678	0.678	0.068	0.551
LipidBot (ours)	0.733	**0.919**	**0.919**	**0.092**	0.778

*Note*: Bold values indicate best result in each category.

Although BM25 achieved higher ranking scores (MRR@10 = 0.809; nDCG@10 = 0.827), this advantage is attributable to the identifier‐heavy nature of the benchmark. Many queries include highly specific gene or protein names (e.g., FAD2, WRI1) that directly match indexed literature, inherently favoring exact lexical matching.

In contrast, within this curated benchmark setting, LipidBot demonstrates greater robustness by achieving superior retrieval coverage (Hit Rate@10 = 0.919) while maintaining competitive ranking performance (MRR@10 = 0.733; nDCG@10 = 0.778). Because its RAG‐based architecture is less dependent on exact terminology alone, LipidBot better accommodates synonymy and semantically varied NL expressions.

Given that each query corresponds to a single gold‐standard document, the maximum achievable Precision@10 is 0.100. LipidBot's Precision@10 of 0.092 approaches this upper bound, confirming consistent prioritization of relevant documents and supporting its utility for discovery‐oriented biological queries.

#### NL‐to‐Cypher generation evaluation

Performance on NL‐to‐Cypher translation was evaluated using two complementary metrics: Execution Success Rate and Result‐Equivalent Correctness. Execution Success Rate measures the percentage of generated Cypher queries that are successfully executed without syntax or runtime errors, reflecting structural validity and executability. Result‐Equivalent Correctness evaluates whether the execution result of a generated query is semantically equivalent to that of the reference query, independent of query formulation or how the results are ordered. All methods were implemented using the same underlying LLM backbone, GPT‐OSS‐20B, to ensure a fair comparison of system design.

Under this evaluation framework, LipidBot achieved an Execution Success Rate of 97.5%, with all generated Cypher queries executing successfully. Approximately 7% of queries generated by CypherQAChain were invalid and failed to execute. For semantic accuracy, LipidBot achieved a Result‐Equivalent Correctness of 95%, substantially outperforming CypherQAChain (69%). These results demonstrate that LipidBot provides more structurally reliable and semantically accurate translation from NL into executable graph queries, thereby improving the robustness and stability of graph‐based biological knowledge access.

## DISCUSSION

FatPlants 2.0 addresses a critical gap in plant lipid biology by integrating fragmented knowledge across species, databases, and literature into a unified, query‐accessible framework. By modeling pathways, genes, enzymes, compounds, species, and orthologs as interconnected entities within a curated knowledge graph, the platform supports exploration across multiple biological relationships rather than static browsing of isolated database records. This structure addresses the relational complexity of metabolic biology, where biological interpretation often depends on synthesizing evidence across pathways, species, and experimental contexts.

When paired with LipidBot, an AI‐assisted natural language interface, this framework enables database‐grounded knowledge access. In this context, database‐grounded means that natural language questions can be linked to structured FatPlants records, graph‐based pathway relationships, and supporting literature. This linkage distinguishes LipidBot from general tools that return publication records or generate broad biological summaries.

Quantitative evaluation further supports the effectiveness of LipidBot for the defined retrieval and graph‐query tasks. LipidBot's hybrid architecture outperformed baselines and general NL‐to‐Cypher frameworks. Its 95% result‐equivalent correctness in Cypher generation indicates that domain‐specific schema grounding and query optimization improve the reliability of graph‐based access to curated plant lipid knowledge. LipidBot also achieved a substantially higher Hit Rate@10 than PubMedBERT (0.919 versus 0.678), suggesting that general biomedical language models may not fully capture the specialized terminology and semantic relationships in plant metabolism literature.

Several limitations warrant consideration. First, although the benchmark provides a controlled evaluation of LipidBot's retrieval and graph‐query functions, it may not fully capture how researchers may use the system in practice. Benchmark questions use fixed, expert‐curated reference answers. However, in practical use, researchers may begin with a candidate gene, an observed lipid phenotype or a preliminary biological hypothesis and then refine their questions through follow‐up queries. Because each LipidBot response is generated from a retrieved subset of relevant literature, broad questions may require iterative refinement. Future evaluation using real‐world user queries will help assess performance under these more exploratory usage conditions.

Second, the current data coverage of FatPlants 2.0 remains incomplete. Although the knowledge graph contains over 10 700 relationships, coverage is still limited for non‐focal species beyond the four primary organisms with curated pathway data. Expanding pathway coverage to additional agronomically important species is therefore a priority. FatPlants 2.0 should also be viewed as a plant lipid‐focused knowledge platform rather than an exhaustive collection of all lipid‐related resources. Several valuable external resources, including species‐specific resources such as CamRegBase (Gomez‐Cano et al., 2020) and broader lipidomics platforms, such as LIPID MAPS, are not currently incorporated. Future development could strengthen the platform not only by adding more databases but also by integrating data types and software tools that better connect genes, lipids, pathways, regulation, and phenotypes.

Long‐term maintenance is essential for FatPlants 2.0 as a community‐facing resource. The platform is hosted on cloud‐based infrastructure and uses a modular architecture that allows the database, literature corpus, graph representation, retrieval system, and AI interface to be updated independently. The AI component is maintained as a replaceable module, allowing newer language models and retrieval strategies to be incorporated while preserving the curated knowledge base and graph structure. Future updates will expand species and pathway coverage, refresh the literature corpus, add new experimental datasets, and cross‐link complementary resources where appropriate. Versioned data releases are planned to improve transparency and reproducibility.

In summary, FatPlants 2.0 demonstrates how LLM‐based querying can be integrated with structured scientific databases. By linking curated plant lipid records, pathway relationships, and literature evidence, the platform helps address data fragmentation and improves access to knowledge of plant lipid metabolism.

## METHODS

### System architecture

**Figure 5:**
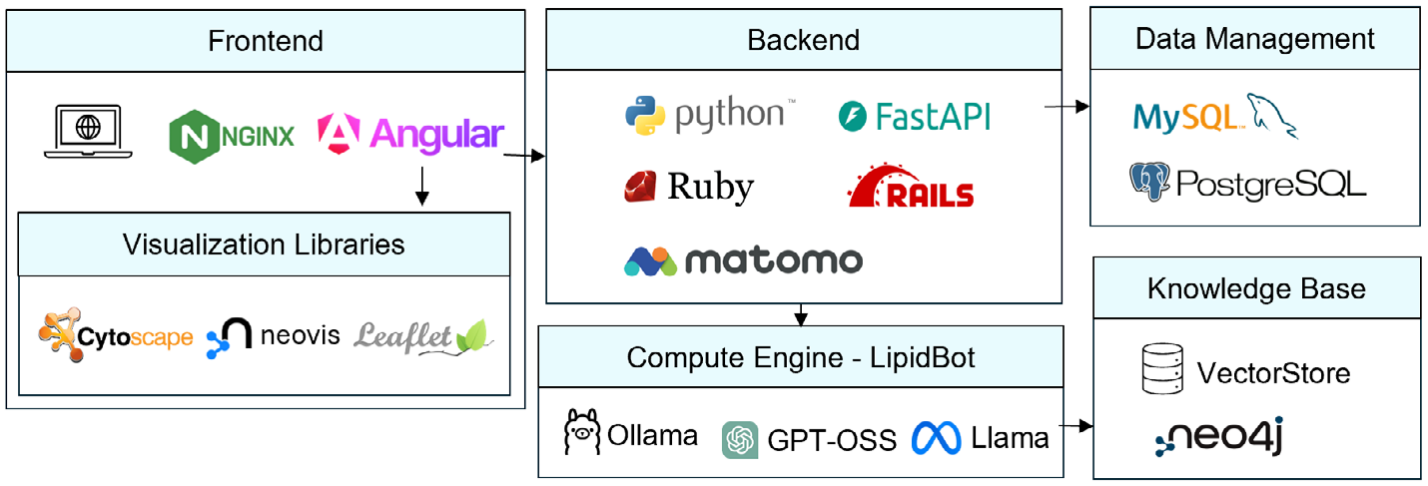
Overview of the full‐stack architecture underlying the platform.

FatPlants is a multimodal AI system that integrates biological databases with NL processing to enable intelligent information retrieval (Figure 5). It employs three‐tier architecture: An Angular frontend featuring specialized biological visualizations; a FastAPI/Rails backend providing API services; and a hybrid data layer combining Neo4j graph database (pathway graph data), relational databases, and vector stores (literature embeddings). NL queries are handled by an LLM‐powered engine, leveraging models such as GPT‐OSS (OpenAI et al., 2025) or LLaMA (Touvron et al., 2023). Additionally, Matomo is used to monitor and analyze user visits and interactions.

### LLM backbone selection

To identify the optimal generative backbone for LipidBot, we benchmarked seven candidate models on a curated set of 40 question–answer pairs spanning fact retrieval, mechanistic reasoning, cross‐species biological inference, and hallucination detection (Methods S1, Tables S4–S6). GPT‐OSS‐20B (OpenAI et al., 2025) achieved the highest overall accuracy (~66%), outperforming mid‐tier models including Vicuna‐7B (~54%), LLaMA3‐8B (~50%), and Mistral‐7B (~46%), and was selected as the LipidBot backbone (Figures S2–S6).

### LipidBot

**Figure 6:**
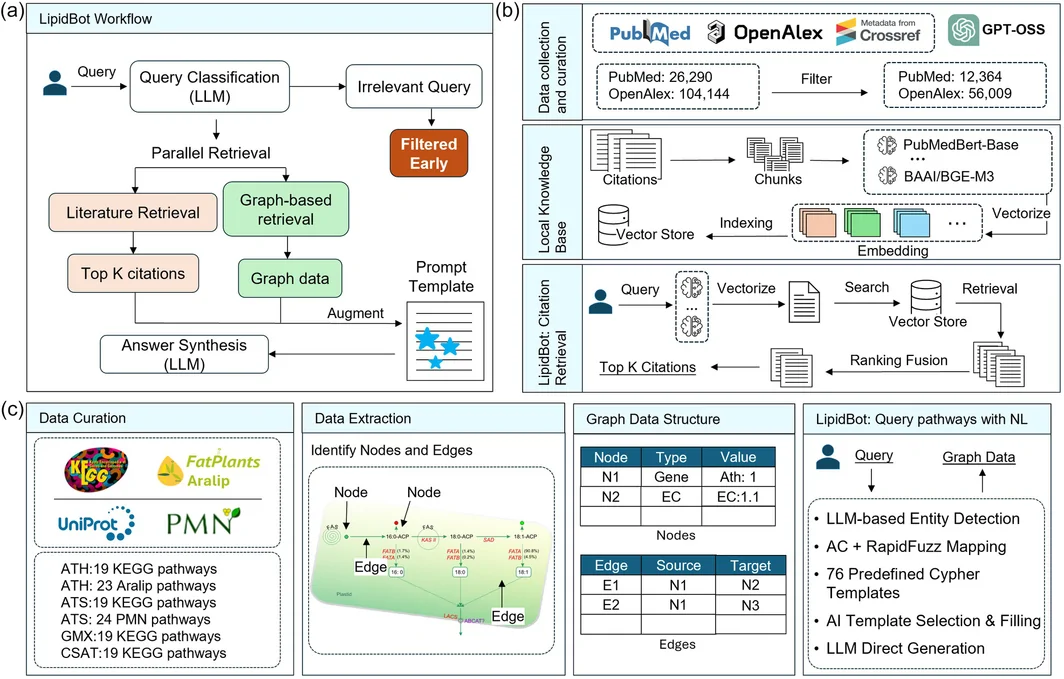
LipidBot System overview. (a) Overall workflow of LipidBot from query classification to database‐grounded evidence retrieval and source‐attributed response synthesis. (b) Literature retrieval workflow, illustrating citation collection, embedding‐based vector scoring, and hybrid citation retrieval in response to user queries. (c) Pathway processing workflow, showing how pathway images are converted into graph representations to support NL‐to‐Cypher querying.

LipidBot serves as the natural language query endpoint for plant lipid biology (Figure 6a). Upon receiving a query, the system first determines whether the question is relevant to plant lipid biology and whether graph‐based retrieval is required; irrelevant queries are filtered early with an informative response. For relevant queries, two retrieval streams run in parallel. When pathway‐level information is needed, a Cypher query is generated and executed against the Neo4j knowledge graph. Concurrently, literature retrieval is performed using a hybrid pipeline that combines dense vector embeddings with BM25 lexical indexing, and ranked results are merged using reciprocal rank fusion (RRF) to return the top 10 citations (Figure 6b). Results from both streams are integrated into a unified context, and a synthesis LLM generates a response with source attribution and explicit uncertainty acknowledgment. In this way, LipidBot provides database‐grounded responses: The answer is not generated solely from the language model's internal knowledge, but is based on retrieved FatPlants records, pathway relationships, and linked literature citations.

#### Citation retrieval

Plant lipid‐related literature was collected from PubMed and OpenAlex using systematic keyword searches (Table S2), yielding 130 434 candidate papers. Relevance filtering was performed using a two‐stage LLM‐based classification pipeline. Stage 1 identified plant‐focused studies with species annotations, and Stage 2 assessed lipid relevance by detecting studies of fatty acids, lipids, or related processes. Papers satisfying both criteria and meeting the predefined confidence threshold were retained, resulting in a curated corpus of over 57 000 articles for retrieval‐augmented generation. To validate classification accuracy, 200 retained papers were randomly sampled and independently classified using Gemini 2.5 Pro, achieving 99.5% agreement for both plant focus and lipid relevance.

Citation retrieval was implemented using a hybrid search strategy that integrates sentence embedding‐based semantic similarity with BM25 lexical indexing. Six embedding models were evaluated using Recall@K across representative queries, and the top‐performing models were selected for deployment (Tables S8 and S9). Vector representations of curated publications were stored in a vector database, while the same corpus was indexed using BM25. For each query, candidate documents were retrieved independently from the embedding models and BM25 index, and the ranked lists were merged using RRF to generate the final citation set. Detailed results of embedding model selection and retrieval parameters are provided in Methods S2.

#### Natural language pathway querying

Our database contains over 120 pathway graphs stored in Neo4j graph database (Tables S10 and S11), representing 110+ unique pathways after deduplication. The graph model consists of nodes representing pathway images, reactions, enzymes, compounds, orthologs, and genes, with interactions encoded as distinct relationship types (Figure S7). In total, the database contains more than 4300 nodes and 10 700 relationships. Entity representation was standardized using KEGG identifiers as primary keys (Kanehisa, 2000).

Because users often submit queries using enzyme, compound, gene, or pathway names rather than database identifiers, these mentions must be mapped to their corresponding identifiers. To achieve this, LipidBot integrates LLM‐based entity extraction with dictionary and fuzzy matching. Candidate entity mentions are first extracted from natural language queries and then matched against a curated plant lipid lexicon using the Aho–Corasick (Aho & Corasick, 1975) string‐matching algorithm, supplemented by RapidFuzz (Ye et al., 2021) to account for lexical variants and minor misspellings. Matched entities are normalized to database identifiers before graph querying.

For Cypher query generation, we used a hybrid template‐based approach. The system maintains 76 predefined Cypher templates covering common query patterns, including shortest‐path and subgraph retrieval. For each query, the LLM selects the most appropriate template and fills it with the resolved entity identifiers. When no template matches the query structure, the LLM generates Cypher directly using the natural language question, extracted entities, graph schema, and template library as context (Figure 6c). Additional details on fuzzy‐matching parameters, lexicon construction, and Cypher template examples are provided in Methods S3.

## CONFLICT OF INTEREST

The authors declare no competing interests.

## Supporting information


Supplemental Methods S1–S3.
**Table S1.** Extended comparison of FatPlants 2.0 with FatPlants 1.0 and related plant lipid resources mentioned in the Introduction.
**Table S2.** Systematic search keywords used to retrieve literature from PubMed and OpenAlex.
**Table S3.** Platform usage statistics (July–December 2025).
**Table S4.** Citation category.
**Table S5.** LLM model configurations and efficiency characteristics.
**Table S6.** Plant lipid LLM benchmark questions.
**Table S7.** The output results. Each question runs five times.
**Table S8.** Ten representative queries used to evaluate the sentence embedding model.
**Table S9.** Recall@K Performance of Candidate Embedding Models.
**Table S10.** KEGG pathways collected for each species.
**Table S11.** Pathway statistics cross species.
**Table S12.** Benchmark dataset with associated download links.
**Figure S1.** Geographical distribution and regional distribution of visits (July–December 2025).
**Figure S2.** Overall performance of large language models on the plant lipid benchmark. Left: Mean score (0–5) averaged across all questions and repeated runs. Right: Normalized accuracy (%) across models. GPT‐OSS‐20B achieves the highest performance in both mean score and accuracy, while other models show moderate to lower performance with varying degrees of consistency.
**Figure S3.** Fact retrieval performance of large language models across benchmark questions. Scores (0–5) are shown for each model across 10 questions (Q1–Q10), with values representing the number of correct responses out of five repeated runs. GPT‐OSS‐20B consistently outperforms other models across most questions, while other models exhibit moderate accuracy and greater variability in factual recall.
**Figure S4.** Mechanistic reasoning performance of large language models across benchmark questions. Scores (0–5) are shown for each model across 10 questions (Q1–Q10), representing the number of correct responses out of five repeated runs. GPT‐OSS‐20B demonstrates consistently higher and more stable performance across questions, while other models show moderate accuracy with increased variability, highlighting challenges in multi‐step biological reasoning tasks.
**Figure S5.** Cross‐species biological inference performance of large language models across benchmark questions. Scores (0–5) are shown for each model across 10 questions (Q1–Q10), representing the number of correct responses out of five repeated runs. GPT‐OSS‐20B demonstrates consistently higher performance across most questions, while other models exhibit greater variability, reflecting challenges in generalizing biological knowledge across species.
**Figure S6.** Hallucination detection performance of large language models across benchmark questions. Scores (0–5) are shown for each model across 10 questions (Q1–Q10), representing the number of correct responses out of five repeated runs. GPT‐OSS‐20B demonstrates more consistent performance across questions, while other models exhibit greater variability, indicating a higher tendency to generate incorrect or unsupported biological statements.
**Figure S7.** Graph‐based modeling of metabolic pathways.

## Data Availability

The source code for FatPlants 2.0 is freely available under the MIT License at GitHub:
https://github.com/MU‐DBL/fatplants (frontend)
https://github.com/MU‐DBL/fastapi‐fatplants (backend)
https://github.com/MU‐DBL/FatPlants_LipidBot (LipidBot source code, benchmark questions, and evaluation) Comprehensive documentation is provided in the repositories. The web‐based version of FatPlants 2.0 is accessible at https://www.fatplants.net/.
