# Supplementary material for: FatPlants 2.0: an AI‐powered platform integrating plant lipid genes, pathways, and literature
Source: Plant J. 2026 Sep 15;127(5):e71117. doi: 10.1111/tpj.71117 (PMC13578592; doi:10.1111/tpj.71117)
Supplement: Supplementary file 1 [file TPJ-127-0-s001.docx]

**Supplementary** **Materials**

# **Supplemental Methods**

## **S1. LLM backbone benchmarking**

To select the optimal LLM backbone for LipidBot, we benchmarked seven candidate models, i.e., Vicuna-7B (Peng et al., 2023), Mistral-7B (Jiang et al., 2023), LLaMA3-8B (Touvron et al., 2023), Qwen3-32B (A. Yang et al., 2025), Phi-4 (Abdin et al., 2024), WizardLM-13B (C. Xu et al., 2025), and GPT-OSS-20B (OpenAI et al., 2025) (Table S5), on a curated dataset of 40 question–answer pairs spanning four reasoning categories: fact retrieval, mechanistic reasoning, cross-species biological inference, and hallucination detection (Table S6). Models were evaluated under consistent prompting conditions without external retrieval augmentation. Each question was executed five times per model to account for stochastic variability, and performance was assessed using accuracy and mean accuracy across repeated trials (Table S7).

GPT-OSS-20B achieved the highest overall performance, with a mean score of 3.3 out of 5 and an accuracy of ~66%. Mid-tier models including Vicuna-7B (~54%), LLaMA3-8B (~50%), and Mistral-7B (~46%) showed moderate performance, while Qwen3-32B, Phi-4 (~39–41%), and WizardLM-13B (~36%) performed lowest (Figure S2). Performance breakdowns across reasoning categories are provided in Supplementary Figures S3–S6. Based on these results, GPT-OSS-20B was selected as the LipidBot backbone.

## **S2. Citation retrieval**

Six embedding models were evaluated across ten representative plant lipid queries using Recall@K. Based on both Recall@5 and Recall@10 performance, the top three models, S-PubMedBERT-MS-MARCO, PubMedBERT-Base-Embeddings, and BGE-M3, were selected for deployment. Vector representations of all curated publications were stored in a vector database, and the same corpus was indexed using BM25. Candidate document lists retrieved from each embedding model and the BM25 index were merged using reciprocal rank fusion to generate the final top 10 citations.

## **S3. Natural-language pathway querying**

For natural-language pathway querying, candidate entity mentions extracted by the LLM were matched against a curated lexicon of 12,847 plant lipid-related terms using the Aho–Corasick string-matching algorithm. Fuzzy string matching with RapidFuzz was used to account for lexical variants and minor misspellings, with a token-set ratio threshold of 85. Matched entities were normalized to database identifiers before Cypher query generation. For example, acetyl-CoA was represented using the KEGG compound identifier C00024.

The system maintains 76 predefined Cypher templates covering common graph-query patterns, including direct entity lookup, shortest-path retrieval, subgraph retrieval, and relationship traversal among genes, enzymes, compounds, orthologs, reactions, and pathways. When no predefined template matches the query structure, the LLM generates Cypher directly using the natural-language question, extracted entities, graph schema, and template library as context.

# **Supplemental Tables**

Table S1. Extended comparison of FatPlants 2.0 with FatPlants 1.0 and related plant lipid resources mentioned in the Introduction.

| Resource | Multi-species / taxonomic scope | Gene–pathway integration | Lipid / fatty acid structure data | Expression or regulatory genomics | Curated literature corpus / retrieval | Graph database | AI/NL query |
| --- | --- | --- | --- | --- | --- | --- | --- |
| FatPlants 1.0 | Partial; selected plant species | Yes | Yes; PlantFAdb fatty acid entries | No | Limited | No | No |
| FatPlants 2.0 | Yes; focal pathway species plus >9,000 species through PlantFAdb fatty acid records | Yes; genes, enzymes, pathways, and orthologs | Yes; PlantFAdb fatty acid entries integrated | Limited; incorporated experimental datasets | Yes; >57,000 curated publications with retrieval support | Yes; Neo4j knowledge graph | Yes; LipidBot |
| ARALIP | No; Arabidopsis-centered | Yes; curated Arabidopsis lipid genes, enzymes, pathways, and mutants | Not primary focus | Mutant/phenotype information | Supporting references, but no retrieval corpus | No | No |
| PlantFAdb | Yes; >9,000 plant species | No | Yes; fatty acid structures, and composition data | No | Supporting references, but no retrieval corpus | No | No |
| KEGG | Yes; broad multi-organism coverage | Yes; pathway, reaction, enzyme, compound, and gene annotations | Limited; not lipid-structure centered | No | No plant lipid-specific retrieval corpus | No | No plant lipid-specific AI/NL query |
| LIPID MAPS | Broad lipid coverage, not plant-centered | Limited; not gene–pathway centered | Yes; lipid structures, nomenclature, and classification | No | No plant lipid-specific retrieval corpus | No | No plant lipid-specific AI/NL query |
| SwissLipids | Broad lipid biology coverage, not plant-centered | Partial; lipid–reaction–protein links | Yes; lipid structures, classes, reactions, and protein links | No | No plant lipid-specific retrieval corpus | No | No plant lipid-specific AI/NL query |
| MetaCyc | Yes; broad organismal coverage | Yes; pathways, reactions, metabolites, enzymes, and genes | Limited; reaction/metabolite centered | No | No plant lipid-specific retrieval corpus | No | No plant lipid-specific AI/NL query |
| CLAIR | Yes; Arabidopsis and multiple oilseed crops | Yes; lipid metabolism genes and pathway-associated annotations | No | Yes; expression, QTL/eQTL, and co-expression information | References included, but no retrieval corpus | No | No |
| PMN / PlantCyc | Yes; broad plant and algal coverage | Yes; plant pathways, enzymes, reactions, compounds, and genes | Limited; pathway/metabolite centered | Omics visualization support | No plant lipid-specific retrieval corpus | No | No plant lipid-specific AI/NL query |

Note: The table summarizes broad resource design and major functional differences rather than providing an exhaustive inventory of all database functions.

Table S2. Systematic search keywords used to retrieve literature from PubMed and OpenAlex.

| Resource | Search Term |
| --- | --- |
| PubMed | ("Plants"[MeSH Terms] OR "Oilseeds" OR "Arabidopsis" OR "Oryza"[MeSH Terms] OR "Zea mays"[MeSH Terms] OR "Glycine max"[MeSH Terms] OR "Brassica"[MeSH Terms] OR "Helianthus"[MeSH Terms] OR "Elaeis" OR "Olea"[MeSH Terms] OR "Gossypium"[MeSH Terms] OR "Camelina" OR "Nicotiana"[MeSH Terms] OR "Triticum"[MeSH Terms] OR "Hordeum"[MeSH Terms]) AND ("Lipids"[MeSH Terms] OR "Fatty Acids"[MeSH Terms] OR "Glycerides" OR "Phospholipids" OR "Sphingolipids" OR "Sterols" OR "Triglycerides" OR "Diglycerides" OR "Monoglycerides" OR "Cardiolipins" OR "Phosphatidylcholines" OR "Phosphatidylethanolamines" OR "Phosphatidylserines" OR "Phosphatidylinositols" OR "Phosphatidylglycerols" OR "Sulfolipids" OR "Polyketides" OR "Lipid Metabolism" OR "Lipid Transport" OR "Lipid Droplets" OR lipid*[tiab] OR fatty acid*[tiab] OR lipidomics[tiab] OR lipidome[tiab] OR lipogenesis[tiab] OR lipogenic[tiab] OR glycolipids[tiab] OR saccharolipids[tiab] OR monogalactosyldiacylglycerol[tiab] OR digalactosyldiacylglycerol[tiab]) NOT (Animals[MeSH] OR Humans[MeSH] OR Fungi[MeSH] OR Bacteria[MeSH] OR Yeasts[MeSH] OR Algae OR Microorganisms) |
| OpenAlex | (plant OR plants OR oilseed OR arabidopsis OR rice OR maize OR soybean OR brassica OR sunflower OR "oil palm" OR olive OR cotton OR camelina OR tobacco OR wheat OR barley) AND ("fatty acid" OR lipid OR lipids OR lipidomics OR lipidome OR lipogenesis OR lipogenic OR glycerolipids OR galactolipids OR glycolipids OR phospholipids OR sphingolipids OR sterols OR sterolipids OR prenolipids OR saccharolipids OR polyketides OR "membrane lipids" OR phosphatidylcholine OR phosphatidylethanolamine OR phosphatidylserine OR phosphatidylinositol OR phosphatidylglycerol OR cardiolipin OR monogalactosyldiacylglycerol OR digalactosyldiacylglycerol OR MGDG OR DGDG OR sulfolipids OR sulfoquinovosyldiacylglycerol OR triacylglycerol OR triacylglycerols OR triglyceride OR triglycerides OR diacylglycerol OR monoacylglycerol OR "lipid metabolism" OR "lipid biosynthesis" OR "lipid synthesis" OR "lipid catabolism" OR "lipid degradation" OR "lipid storage" OR "lipid transport" OR "lipid trafficking" OR "lipid assembly" OR "lipid accumulation" OR "lipid droplets" OR "lipid bodies" OR "oil bodies" OR "lipid bilayer" OR "lipid membrane" OR "lipid vesicles" OR "lipid composition" OR "lipid profile" OR "lipid analysis" OR "lipid extraction" OR "lipid characterization" OR Monogalactosyldiacylglycerol OR Digalactosyldiacylglycerol) NOT (animal OR human OR mammal OR fungi OR bacteria OR yeast OR algae OR microorganism) |

Table S3. Platform usage statistics (July–December 2025).

| Metric | Value |
| --- | --- |
| Total visits | 2,378 |
| Countries | 55 |
| Returning visitors | 767 (32%) |
| Total interactions | 10,856 |
| Avg. actions per visit | 4.6 |
| Returning user engagement | 8.5 actions, 8.8 min |

Table S4. Citation category.

Citation Category

Fatty Acid Biosynthesis Pathways

Desaturation & Modification

Lipid Storage & Mobilization

Genetic Regulation

Species-Specific Profiles

Metabolic Engineering

Analytical Methods

Subcellular Localization

Table S5**. LLM model configurations and efficiency characteristics.**

| LLM | Price | Context |
| --- | --- | --- |
| Vicuna 7b | $0.20 / $0.20 | N/A |
| Mistral-7b | $0.20 / $0.20 | 32.8K |
| Llama3 – 8b | $0.03 / $0.04 | 8.2K |
| Quen3 -32b | $0.08 / $0.24 | 41K |
| Phi-4 | $0.07 / $0.14 | 16.4K |
| Wizardlm-13b | $0.30 / $0.30 | N/A |
| GPT-OSS 20b | $0.03 / $0.11 | 131.1K |

The table summarizes the evaluated large language models, including their inference costs (input/output pricing) and maximum context window sizes. These parameters provide insights into computational efficiency and scalability, enabling comparison of trade-offs between model performance, cost, and context capacity.

Table S6**. Plant lipid LLM benchmark questions.**

Section 1 - Fact Retrieval

| No. | Question | Answer |
| --- | --- | --- |
| Q1 | Which enzyme catalyzes the final step of triacylglycerol (TAG) synthesis in plants? | Diacylglycerol acyltransferase (DGAT) catalyzes the final step by transferring an acyl group from acyl-CoA to diacylglycerol (DAG) to form TAG. |
| Q2 | Where does de novo fatty acid synthesis occur in plant cells? | Primarily in plastids (chloroplasts). |
| Q3 | What is the function of the FAD2 enzyme in plants? | FAD2 is an omega-6 fatty acid desaturase that converts oleic acid (18:1) into linoleic acid (18:2) in the endoplasmic reticulum. |
| Q4 | What is the primary storage lipid in most oilseed plants? | Triacylglycerol (TAG). |
| Q5 | What cellular organelle stores TAG in plant seeds? | Oil bodies (lipid droplets). |
| Q6 | Which enzyme catalyzes the first committed step of fatty acid synthesis? | Acetyl-CoA carboxylase (ACCase), which converts acetyl-CoA to malonyl-CoA. |
| Q7 | What is the function of oleosin proteins in seeds? | They stabilize oil bodies and prevent lipid droplet fusion. |
| Q8 | Which plant tissue typically accumulates the highest levels of storage oil? | Seeds. |
| Q9 | Which lipid class forms the structural basis of plant cell membranes? | Phospholipids and glycolipids. |
| Q10 | Which pathway converts fatty acids into energy during seed germination? | β-oxidation. |

Section 2 - Mechanistic Reasoning

| No. | Question | Answer |
| --- | --- | --- |
| Q11 | What metabolic change occurs if FAD2 is knocked out in Arabidopsis seeds? | Oleic acid increases while linoleic acid decreases due to reduced desaturation. |
| Q12 | What would happen if DGAT activity increases in developing seeds? | Increased DGAT activity generally increases TAG synthesis and seed oil accumulation. |
| Q13 | What is the effect of strongly reducing ACCase activity in plants? | Fatty acid synthesis decreases, reducing lipid production and potentially impairing growth. |
| Q14 | What happens if β-oxidation is impaired in germinating seeds? | Stored fatty acids cannot be efficiently converted to energy, resulting in poor seedling growth. |
| Q15 | What would likely happen if oleosin expression is reduced in seeds? | Oil bodies may become unstable and lipid droplets may fuse. |
| Q16 | What effect would increase fatty acid elongase activity have? | It would increase production of very-long-chain fatty acids. |
| Q17 | What metabolic consequence might occur if TAG breakdown is inhibited during germination? | Seedlings may suffer energy deficiency and delayed development. |
| Q18 | How might overexpression of WRINKLED1 (WRI1) affect lipid metabolism? | It activates genes involved in fatty acid synthesis, increasing oil accumulation. |
| Q19 | What happens when carbon flux is redirected toward fatty acid synthesis? | More carbon enters lipid biosynthesis, often increasing seed oil content. |
| Q20 | What might occur if membrane lipid desaturation decreases? | Membrane fluidity decreases, which can affect stress tolerance and cellular function. |

Section 3 - Cross-Species Biological Inference

| No. | Question | Answer |
| --- | --- | --- |
| Q21 | Would soybean likely have an enzyme similar to Arabidopsis FAD2? | Yes. Fatty acid desaturation pathways are conserved and soybean contains FAD2 homologs. |
| Q22 | Why might oil accumulation strategies differ between Arabidopsis and maize? | Because of differences in seed structure, metabolic regulation, and genetic networks. |
| Q23 | Could genes that increase oil in Arabidopsis work in other oilseed crops? | Possibly, but species-specific metabolic regulation must be considered. |
| Q24 | Why might lipid metabolism differ between leaves and seeds? | Leaves focus on membrane lipids for photosynthesis while seeds accumulate TAG for storage. |
| Q25 | Would algae likely share fatty acid synthesis pathways with plants? | Yes, many algae share similar fatty acid synthesis mechanisms. |
| Q26 | Could engineering TAG biosynthesis increase oil in vegetative tissues? | Yes, but metabolic balance must be managed to avoid growth defects. |
| Q27 | Why might oil accumulation differ between tropical and temperate plants? | Due to environmental adaptation and differences in membrane lipid composition. |
| Q28 | Would FAD3 likely exist in other oilseed crops? | Yes. FAD3 homologs are common and produce linolenic acid (18:3). |
| Q29 | Could increasing oil in crops affect seed germination? | Yes, because altered lipid metabolism may influence energy mobilization. |
| Q30 | Why must metabolic engineering consider whole-pathway regulation? | Changing one enzyme can disrupt metabolic balance and carbon allocation. |

Section 4 - Hallucination Detection

| No. | Question | Answer |
| --- | --- | --- |
| Q31 | What is the function of the Arabidopsis enzyme LIPX1? | No enzyme named LIPX1 is documented in Arabidopsis lipid metabolism. |
| Q32 | How does TAGF3 regulate fatty acid elongation? | There is no known plant gene or protein called TAGF3. |
| Q33 | What pathway does FADX7 regulate in plant lipid metabolism? | There is no documented enzyme named FADX7. |
| Q34 | What role does OILR2 kinase play in seed lipid biosynthesis? | There is no known OILR2 kinase involved in plant lipid metabolism. |
| Q35 | How does PLASTLIP3 regulate plastid lipid synthesis? | No such enzyme PLASTLIP3 is described in the literature. |
| Q36 | What is the function of SEEDTAG9 in oil accumulation? | There is no gene named SEEDTAG9 in plant lipid research. |
| Q37 | How does LIPOX regulator LPX10 affect fatty acid biosynthesis? | LPX10 is not a known regulator in plant lipid metabolism. |
| Q38 | What metabolic role does FATB3 enzyme play in Arabidopsis? | FATB enzymes exist but FATB3 specifically is not documented. |
| Q39 | How does TAG kinase TGK1 regulate lipid storage? | There is no evidence of a TAG kinase called TGK1. |
| Q40 | What function does Arabidopsis lipid gene LPDX5 perform? | No gene named LPDX5 is reported in plant lipid metabolism. |

The table lists 40 curated questions organized into four categories: fact retrieval (Q1–Q10), mechanistic reasoning (Q11–Q20), cross-species biological inference (Q21–Q30), and hallucination detection (Q31–Q40). These questions are designed to evaluate model performance in knowledge recall, causal reasoning, cross-system generalization, and robustness against hallucinated or unsupported biological information.

Table S7**.** The output results. Each question runs five times.

| Fact Retrieval | Vicuna 7b | Mistral-7b | Llama3 – 8b | Quen3 -32b | Phi-4 | Wizardlm-7b | GPT-OSS 20b |
| --- | --- | --- | --- | --- | --- | --- | --- |
| Q1 | 2 | 2 | 4 | 2 | 3 | 2 | 4 |
| Q2 | 2 | 4 | 3 | 2 | 2 | 0 | 3 |
| Q3 | 3 | 2 | 2 | 3 | 2 | 2 | 3 |
| Q4 | 3 | 1 | 2 | 2 | 2 | 3 | 4 |
| Q5 | 2 | 3 | 2 | 2 | 1 | 2 | 5 |
| Q6 | 3 | 2 | 2 | 1 | 3 | 1 | 4 |
| Q7 | 4 | 2 | 3 | 2 | 2 | 2 | 3 |
| Q8 | 3 | 2 | 3 | 1 | 2 | 1 | 4 |
| Q9 | 2 | 3 | 2 | 2 | 2 | 2 | 4 |
| Q10 | 3 | 2 | 2 | 2 | 2 | 1 | 2 |
| Mechanistic Reasoning | Vicuna 7b | Mistral-7b | Llama3 – 8b | Quen3 -32b | Phi-4 | Wizardlm-7b | GPT-OSS 20b |
| Q1 | 3 | 2 | 3 | 2 | 2 | 1 | 4 |
| Q2 | 2 | 3 | 2 | 1 | 2 | 2 | 4 |
| Q3 | 3 | 1 | 2 | 3 | 1 | 1 | 3 |
| Q4 | 2 | 3 | 3 | 2 | 2 | 2 | 2 |
| Q5 | 3 | 2 | 3 | 2 | 3 | 1 | 4 |
| Q6 | 3 | 3 | 2 | 2 | 1 | 2 | 3 |
| Q7 | 3 | 2 | 3 | 1 | 2 | 3 | 3 |
| Q8 | 2 | 3 | 2 | 1 | 2 | 3 | 4 |
| Q9 | 4 | 3 | 3 | 3 | 2 | 2 | 3 |
| Q10 | 3 | 2 | 2 | 3 | 1 | 2 | 3 |
| Cross-Species Biological Inference | Vicuna 7b | Mistral-7b | Llama3 – 8b | Quen3 -32b | Phi-4 | Wizardlm-7b | GPT-OSS 20b |
| Q1 | 3 | 3 | 2 | 2 | 2 | 2 | 4 |
| Q2 | 3 | 2 | 1 | 2 | 2 | 2 | 4 |
| Q3 | 3 | 2 | 3 | 1 | 1 | 1 | 3 |
| Q4 | 2 | 2 | 3 | 2 | 1 | 1 | 3 |
| Q5 | 3 | 1 | 3 | 1 | 2 | 2 | 3 |
| Q6 | 2 | 1 | 2 | 2 | 0 | 2 | 2 |
| Q7 | 4 | 2 | 3 | 2 | 0 | 1 | 4 |
| Q8 | 2 | 2 | 3 | 1 | 1 | 2 | 3 |
| Q9 | 3 | 2 | 2 | 1 | 2 | 1 | 2 |
| Q10 | 3 | 1 | 2 | 2 | 2 | 1 | 4 |
| Hallucination Detection | Vicuna 7b | Mistral-7b | Llama3 – 8b | Quen3 -32b | Phi-4 | Wizardlm-7b | GPT-OSS 20b |
| Q1 | 2 | 3 | 3 | 2 | 2 | 1 | 4 |
| Q2 | 3 | 3 | 3 | 2 | 2 | 1 | 3 |
| Q3 | 4 | 2 | 2 | 2 | 0 | 2 | 2 |
| Q4 | 2 | 1 | 2 | 1 | 2 | 2 | 4 |
| Q5 | 2 | 3 | 2 | 1 | 3 | 2 | 3 |
| Q6 | 3 | 2 | 3 | 2 | 2 | 3 | 4 |
| Q7 | 2 | 2 | 3 | 3 | 3 | 1 | 3 |
| Q8 | 2 | 2 | 2 | 2 | 2 | 2 | 3 |
| Q9 | 3 | 2 | 0 | 2 | 3 | 1 | 3 |
| Q10 | 3 | 2 | 2 | 2 | 2 | 1 | 2 |

Table S8. Ten representative queries used to evaluate the sentence embedding model.

| Target PMID | Question |
| --- | --- |
| 11814526 | Which Arabidopsis mutants were assessed to understand membrane lipid formation? |
| 21081663 | In germinating oilseed plants, what type of enzyme is involved in breaking down triglycerides? |
| 21081663 | Which enzyme is related to the formation of 13-LOX-derived lipid peroxides in oil body membranes? |
| 29813189 | How do the lipid classes in epidermal bladder cells (EBC) respond to salt stress? |
| 26748916 | Tell me about the role of acylcarnitines in plant lipid metabolism |
| 29873878 | In which sorghum leaf cell type did lipid droplets primarily accumulate |
| 29873878 | Which genes were experimented with to increase triacylglycerol accumulation in plant leaves? |
| 38772323 | Any transcription factor is known to regulate EgFATA expression and promotes oleic acid accumulation in oil palm. |
| 20002588 | Which enzymes were identified as responsible for plastidial enolase and phosphoglyceromutases activities in developing Arabidopsis embryos? |
| 15078327 | What phenotypic effects were observed in sterol pathway mutants in Arabidopsis? |

Table S9. Recall@K Performance of Candidate Embedding Models.

| Model | Recall@5 | Recall@10 |
| --- | --- | --- |
| pritamdeka/S-PubMedBert-MS-MARCO | 0.9 | 0.9 |
| NeuML/pubmedbert-base-embeddings | 0.8 | 0.9 |
| BAAI/bge-m3 | 0.8 | 0.9 |
| michiyasunaga/BioLinkBERT-base | 0.5 | 0.6 |
| cambridgeltl/SapBERT-from-PubMedBERT-fulltext | 0.6 | 0.6 |
| pritamdeka/S-BioBert-snli-multinli-stsb | 0.5 | 0.6 |

Performance of six embedding models on a test set of 10 representative questions (Table S4). Recall@K measures the proportion of queries for which the correct papers appeared among the top K retrieved results. The top three models were selected for production.

Table S10. KEGG pathways collected for each species.

KEGG

Fatty acid biosynthesis

Fatty acid elongation

Fatty acid degradation

Steroid biosynthesis

Valine, leucine and isoleucine degradation

Lysine degradation

Other glycan degradation

Glycerolipid metabolism

Glycerophospholipid metabolism

Ether lipid metabolism

Arachidonic acid metabolism

Linoleic acid metabolism

alpha-Linolenic acid metabolism

Sphingolipid metabolism

Glycosphingolipid biosynthesis

Brassinosteroid biosynthesis

Biosynthesis of unsaturated fatty acids

Fatty acid metabolism

RNA degradation

Table S11. Pathway statistics cross species.

| Species | Number of pathways | Source |
| --- | --- | --- |
| ath | 19+23 | KEGG+ARALIP |
| ats | 19 | KEGG |
| gmx | 19 | KEGG |
| csat | 19 | KEGG |

Table S12. Benchmark dataset with associated download links

| 800-question Question-to-Citation benchmark | https://github.com/MU-DBL/FatPlants_LipidBot/blob/main/file/categoried_citation_sampled_800_papers.csv |
| --- | --- |
| 393 NL-to-Cypher generation benchmark | https://github.com/MU-DBL/FatPlants_LipidBot/blob/main/file/pathway_evaluation_question.csv |

# **Supplemental Figures**

Figure S1. Geographical distribution and regional distribution of visits (July–December 2025).

Figure S2**. Overall performance of large language models on the plant lipid benchmark.** Left: Mean score (0–5) averaged across all questions and repeated runs. Right: Normalized accuracy (%) across models. GPT-OSS-20B achieves the highest performance in both mean score and accuracy, while other models show moderate to lower performance with varying degrees of consistency.

Figure S3**. Fact retrieval performance of large language models across benchmark questions.** Scores (0–5) are shown for each model across 10 questions (Q1–Q10), with values representing the number of correct responses out of five repeated runs. GPT-OSS-20B consistently outperforms other models across most questions, while other models exhibit moderate accuracy and greater variability in factual recall.

Figure S4. **Mechanistic reasoning performance of large language models across benchmark questions.** Scores (0–5) are shown for each model across 10 questions (Q1–Q10), representing the number of correct responses out of five repeated runs. GPT-OSS-20B demonstrates consistently higher and more stable performance across questions, while other models show moderate accuracy with increased variability, highlighting challenges in multi-step biological reasoning tasks.

Figure S5**. Cross-species biological inference performance of large language models across benchmark questions.** Scores (0–5) are shown for each model across 10 questions (Q1–Q10), representing the number of correct responses out of five repeated runs. GPT-OSS-20B demonstrates consistently higher performance across most questions, while other models exhibit greater variability, reflecting challenges in generalizing biological knowledge across species.

Figure S6**. Hallucination detection performance of large language models across benchmark questions.** Scores (0–5) are shown for each model across 10 questions (Q1–Q10), representing the number of correct responses out of five repeated runs. GPT-OSS-20B demonstrates more consistent performance across questions, while other models exhibit greater variability, indicating a higher tendency to generate incorrect or unsupported biological statements.

Figure S7. Graph-Based Modeling of Metabolic Pathways
